# Risk factors and significance of post-operative edema in Parkinson Disease patients submitted to deep brain stimulation. A ten-year case series

**DOI:** 10.1007/s10072-024-07774-4

**Published:** 2024-09-19

**Authors:** Alessandro Izzo, Francesco Bove, Quintino Giorgio D’Alessandris, Danilo Genovese, Tommaso Tufo, Manuela D’Ercole, Giovanni Pennisi, Federica Figà, Marco Obersnel, Valerio Perotti, Maria Filomena Fuggetta, Anna Rita Bentivoglio, Paolo Calabresi, Alessandro Olivi, Carla Piano, Nicola Montano

**Affiliations:** 1 Department of Neurosurgery, Fondazione Policlinico Universitario A. Gemelli IRCCS, Rome, Italy; 2 Department of Neurology, Fondazione Policlinico Universitario A. Gemelli IRCCS, Rome, Italy; 3 Università Cattolica del Sacro Cuore, Largo Francesco Vito, 1, Rome, 00168 Italy; 4 Department of Anesthesiology, Fondazione Policlinico Universitario A. Gemelli IRCCS, Rome, Italy

**Keywords:** Asleep, Deep brain stimulation, Directional electrode, Edema, Microelectrode recording, Parkinson disease

## Abstract

**Background:**

Peri-electrode edema after deep brain stimulation (DBS) surgery for Parkinson Disease (PD) has been reported in up to 100% of cases. The clinical significance of this finding is unclear, with most papers suggesting a benign course. The risk factors are also poorly defined. We aimed at defining the incidence rate, the clinical significance and the predictive factors of peri-electrode edema in patients undergoing DBS for PD.

**Methods:**

We reviewed data of 119 patients treated with frameless stereotactic DBS for PD between 2012 and 2022 at our Institution. A mixed-technique targeting was adopted. Awake surgery was used in 64.7% cases; in most cases, microelectrode recording (MER) was adopted. The target was the subthalamic nucleus (STN) in 91.2% cases.

**Results:**

Ninety patients were included. Postoperative edema related to lead placement was noticed in 40% patients after a median time of 2 days since surgery; in 88.9% of these cases, it was limited to subcortical white matter. Symptomatic edema was registered only in one case (1.1%), confirming previous reports on the benign clinical course. The only independent predictive factor for edema onset was asleep surgery (*p* = 0.0451). Notably, the use of directional electrodes was not associated with an increased risk of edema at multivariable analysis. Clinical parameters including age, and timing of CT scanning, did not affect edema onset.

**Conclusions:**

We confirmed the very low rate of symptomatic edema in DBS for PD. When feasible, awake DBS using MER is the ideal technique to reduce the risk of radiologic postoperative edema.

**Supplementary Information:**

The online version contains supplementary material available at 10.1007/s10072-024-07774-4.

## Introduction

The occurrence of peri-electrode edema after deep brain stimulation (DBS) surgery for Parkinson Disease (PD) was first reported in 2004 [1]. The causes of such phenomenon are not well recognized. The available studies supported multiple potential mechanisms to explain its onset: surgical microtraumatism because of lead insertion, cortical or subcortical venous infarction, microinflammatory environment involving inflammatory cells and secreted cytokines, or immunologic mechanisms as a response to the implantation material [2]. Notwithstanding it is currently a well-recognized event, the incidence of postoperative edema after DBS varies widely in literature between < 1% and 100% of cases [1, 3–15]. Such a wide range has been associated to several factors, namely the definition of edema (purely radiologic occurrence vs. symptomatic cases), the radiological technique used to assess edema onset (MRI vs. CT) and the timing of post-DBS scan. Similarly, no agreement exists on the risk factors for the occurrence of such edema, though the clinical course is generally reported as benign and long-term sequelae are not described.

In the present work, we aimed at assessing the incidence of radiological and symptomatic edema after DBS surgery for PD at a large academic center. We explored the risk factors and the clinical implications of edema occurrence in our patients, and we put our findings in the context of the available literature.

## Materials and methods

### Patients selection

We retrospectively evaluated a case series of 119 patients treated with DBS for PD between 2012 and 2022 at the Department of Neurosurgery of our Institution. PD was diagnosed using the United Kingdom Parkinson’s Disease Brain Bank criteria. We adopted inclusion and exclusion criteria proposed by the core assessment program for surgical interventional therapies in Parkinson’s disease panel (CAPSIT-PD) [16]. The study was conducted according to the principles set forth in the Declaration of Helsinki and was approved by our Institutional Ethics Committee (Prot. No. 4578). Patients signed an informed consent according to the institutional research proposals. This paper was drafted according to the Preferred Reporting Of CasE Series in Surgery (PROCESS) Guidelines [17], as far as applicable.

### Surgical technique

All patients were operated using a stereotactic frameless technique (Nexframe™, Medtronic) [18]. The preoperative planning was made on StealthStation Surgical Navigation System (S7 Medtronic^®^ before 2017, S8 Medtronic^®^ since 2017). A mixed-technique targeting, in which atlas-based stereotactic coordinates were adjusted based on direct target visualization on MRI, was adopted. O-Arm was used since 2017 to allow for fiducials-less registration and intraoperative targeting confirmation [19]. LeadPoint (FHC Inc, Bowdoin, ME, USA) microelectrodes recording (MER) were used in all cases operated with awake surgery and in 75% cases operated with asleep surgery. Single-track multipass registration technique was adopted [18]. STN or Globus pallidus interna (GPi) were identified according the previously published neurophysiological criteria [20, 21]. Briefly, STN was distinguished from the dorsally located zona incerta and lenticular fasciculus (field H2) by a sudden increase in background noise level and was identified by the presence of neurons with characteristic 25- to 45-Hz firing rates and irregular firing patterns, with tremor-related activity and modulated by passive movements; the ventral border of the STN was recognized by a decrease of background noise and a decrease of multi-unit activity, with more regular firing units of the substantia nigra pars reticulata. On the other hand, GPi was distinguished from the medial medullary lamina by an increase of multi-unit firing activity and was identified by a tonic regular discharge frequency between 5 and 30 Hz; the ventral border of the GPi was recognized by a sudden decrease in background noise and multi-unit activity, due to reaching the optic tract. Quadripolar implanted electrodes were: Vercise™ (Boston Scientific, Marlborough, MA, USA), 3389 lead (Medtronic Inc, Minneapolis, MN, USA) and Abbott/St Jude directed 6172 (Abbott laboratories, Abbott Park, IL, USA).

### Anesthetic technique

An advice on awake vs. asleep surgery was performed by the multidisciplinary team based on patients’ clinical conditions, compliance and neuropsychiatric assessment. The final decision was made jointly with the patient.

In this work, we have defined as «awake surgery» a surgery performed under conscious sedation, using short-acting drugs which are stopped before the recordings and testing. This approach allows optimal conditions for MER or stimulation testing. Dexmedetomidine is a central-acting highly selective α2-adrenergic receptor agonist that offers sedation and anxiolysis and helps maintain hemodynamic stability with an anesthetic-sparing effect. Dexmedetomidine is particularly valuable when eloquent areas of the brain are stimulated and an awake, cooperative patient is required for neurocognitive testing [22]. We use Dexmedetomidine by infusion at 1 mcg/kg/h for 20 min as a loading dose, followed by a dosage 0.5–0.6 mcg/kg/h up to MER recording. All patients had spontaneous ventilation with supplemental oxygen (2 L/min) through a nasal prong; no laryngeal mask or other devices were used. The depth of anesthesia was monitored using bispectral index (BIS). Elias et al. found that the subthalamic MER signals were equivalent to the awake state when sedation was titrated to an easily arousable state (BIS value > 70) in patients with Parkinson’s disease [23]. Before the start of surgery, a bilateral scalp block was done to provide effective analgesia during awake DBS.

In case of asleep surgery, a totally intravenous anesthesia (TIVA) protocol with TCI (Target Controlled Infusion) of both propofol and remifentanil, and bilateral scalp block as already described, was used. After premedication with oral midazolam, induction was performed using remifentanil 2–3 ng/ml and propofol 3–3.5 µg/ml for 5 min based on Schnider’s pharmacokinetic model [24, 25]. Anesthesia was maintained with remifentanil 4 ng/ml and propofol 2.5–3.0 µg/ml according to patients’ physiological parameters and BIS monitoring.

### Clinical data collection and radiological evaluation

We analyzed baseline clinical characteristics of patients, including duration of PD symptoms. Moreover, the presence of comorbidities was registered. In detail, we focused on arterial hypertension, diabetes, autoimmune diseases (with a formal allergological or rheumatologic diagnosis) and vascular diseases, including arteritis, amyloid angiopathy and thrombophilia.

Post-surgical edema was assessed on the post-operative CT scan performed by 7 days after surgery. Post-operative images were independently reviewed by two Authors (A.I. and Q.G.D.); uncertainties or disagreements were solved by discussion with a third Author (N.M.). We excluded from analysis for excess of artifacts those CT scans in which more than two contiguous slices, spaced 1.25 mm or more, were not interpretable. We registered the side of edema, its maximum diameter, its location, the presence of associated symptoms, the need of medical therapy, and the presence of brain hemorrhage. The following symptoms were considered as related to peri-lead edema: severe headache, confusion, focal neurological deficits, seizures. A mild hypodensity limited to the insertion site of the electrode was not considered as edema, as it can be attributed to the cauterization of the cortical surface routinely performed before lead insertion.

### Statistical analysis

Continuous variables were described using mean ± standard deviation (SD) or median and range. Comparison of continuous variables between groups was performed using the Mann-Whitney U test; confidence interval for median difference was calculated using the Hodges-Lehmann method. Categorical variables were compared using Chi-square statistic, applying the Fisher exact test when appropriate. For confidence interval calculation, the Odds Ratio was taken into consideration. Multivariable analysis for predictive factors for edema onset was performed using the logistic regression model, while adjusting for the variables which resulted significant at univariable analysis, namely anesthesia type (awake vs. asleep), implant of directional electrodes, and number of MER tracks. Electrode brand was excluded for collinearity. Analyses were performed using software StatView ver 5.0 (SAS Institute, Cary, NC, USA) and MedCalc ver 20.015 (MedCalc Software Ltd., Ostend, Belgium). A *p* < 0.05 was considered as significant.

## Results

### Patients features

Postoperative CT scan was not available for 17 patients of our series and was scarcely interpretable due to lead artifacts in 12 further patients. The 90 DBS patients for which a postoperative CT scan was available and interpretable thus built our study group.

Baseline characteristics of patients are listed in Online Resource 1, Supplementary Table S1. Mean age was 57.4 ± 10.0 years, and 63.3% of included patients were male. Prevalence of comorbidities was less than 10% of cases, except for arterial hypertension which was registered in 18.9% of cases.

Surgical data are detailed in Table 1. The target was the STN in 91.1% of cases, while surgery was performed on awake patients in 60% cases and was bilateral in 93.3% of patients. Mean number of MER tracks per patient was 2.5; in 31 cases, > 2 MER tracks were performed. Directional electrodes were used in 72 (80%) cases.

**Table 1:** Surgical characteristics of the study patients (*n* = 90)

Feature	*n* (%)
Target	
STN	82 (91.1)
GPi	5 (5.6)
VIM	3 (3.3)
Anesthesia setting	
Awake	54 (60)
Asleep	36 (40)
N electrodes	
1	6 (6.7)
2	84 (93.3)
Directional electrode	72 (80)
Lead Brand	
Boston Scientific	56 (62.2)
Medtronic	18 (20)
Abbott/St Jude	16 (17.2)
*N* MER tracks per patient (mean ± SD)	2.5 ± 0.8
Surgery Time (mean ± SD) (min)	282 ± 86

GPi, internal globus pallidus; MER, microelectrode recording; STN, subthalamic nucleus; VIM, ventro-intermedius nucleus of the thalamus

### Postoperative CT scan

Mean timing of post-operative CT scan was 2.4 ± 1.1 days (range, 1–8). As shown in Table 2, some postoperative edema related to lead placement was detectable in 40% patients. In most cases it was confined in the subcortical white matter (Online Resource 2, Supplementary Fig. S1*left*), while only in 4 cases it was extended to the whole electrode (Online Resource 2, Supplementary Fig. S1*right).* Noteworthy, only in one case some symptoms related to edema were detected. This was a 68-year-old male undergoing bilateral STN DBS who experienced postoperative agitation and confusion, which resolved after some days with mere observation. Edema was bilateral but limited to lead insertion site. In another case, clinical worsening was due to post-operative hemorrhage and not to edema. It was a case of 69-year-old male undergoing bilateral GPi DBS who experienced postoperative reduction of level of consciousness; CT scan showed whole electrode bilateral edema associated with intracerebral hemorrhage requiring emergent craniotomy. Another patient experiencing subcortical unilateral postoperative edema was treated with short-course dexamethasone despite the lack of unambiguous clinical disturbances caused by edema.

**Table 2:** Radiological characteristics of the study patients (*n* = 90)

Feature	*n*	%
Lead Edema		
No	54	60
Yes	36	40
Unilateral	26	28.9
Bilateral	10	11.1
Subcortical only	32	35.6
Whole electrode	4	4.4
Largest edema diameter (mm)	17.5 ± 5.6	
Symptomatic edema	1	1.1
Medical therapy for edema	1	1.1
Hemorrhage		
No	79	87.8
Yes	11	12.2
Symptomatic	1	1.1

### Predictive factors for edema onset

Univariable analysis of clinical and surgical predictive factors for post-operative lead edema is presented in Tables 3 and 4, respectively. Timing of post-operative CT scan did not affect edema diagnosis: rate of positive findings for edema was not different comparing scans performed < 3 days vs. those performed ≥ 3 days after surgery. As concerns clinical features, no significant predictor of edema was identified; arterial hypertension showed a trend towards a reduced edema risk (*p* = 0.0532, Fisher Exact Test). As concerns surgical features, awake DBS (*p* = 0.0002, Fisher Exact Test) and a number of MER tracks ≤ 2 (*p* = 0.0171, Fisher Exact Test) were significantly associated to a reduced edema risk. The use of directional electrodes, instead, was associated with an increased risk of postoperative edema (*p* < 0.0001, Fisher Exact Test). Considering the electrode brands, the highest percentage of edema cases was recorded with Abbott/St Jude leads (*p* = 0.0001, Chi-square test).

**Table 3:** Clinical predictive factors for postoperative edema (univariable analysis)

Parameter	Edema	*p*	95% CI
Timing of postoperative CT scan (days)	2 (1–8) vs. 2 (1–7) in no edema	0.1357^#^	0.0–0.0
Timing of postoperative CT scan		0.1309*	0.4–10.1
< 3 days	25/70 (35.7%)		
≥ 3 days	11/20 (55%)		
Sex		0.6590*	0.5–3.1
Male	24/57 (42.1%)		
Female	12/33 (36.4%)		
Age (y)	60 (18–70) vs. 59 (43–73) in no edema	0.8496^#^	-4.0–4.0
Time since PD onset (y)	10.5 (3–45) vs. 10 (5–35) in no edema	0.9835^#^	-2.0–2.0
Autoimmune disease		0.1378*	0.0–1.6
Yes	1/8 (12.5%)		
No	35/82 (42.7%)		
Diabetes		> 0.9999*	0.1–25.0
Yes	1/2 (50%)		
No	35/88 (39.8%)		
Hypertension		0.0532*	0.1–1.0
Yes	3/17 (17.6%)		
No	33/73 (45.2%)		
Vascular diseases		0.2719*	0.0–4.0
Yes	0/3 (0%)		
No	36/87 (14.4%)		

CI, confidence interval. PD, Parkinson Disease. *, Fisher Exact test. ^#^, Mann-Whitney U test

**Table 4:** Surgical predictive factors for postoperative edema (univariable analysis)

Feature	edema	*p*	95% CI
Target		0.1692^+^	
STN	31/82 (37.8%)		0.0–1.4 (STN vs. GPi)
Gpi	4/5 (80%)		0.0-3.2 (GPi vs. VIM)
VIM	1/3 (33.3%)		0.1–9.5 (STN vs. VIM)
Anesthesia		0.0002*	0.1–0.5
Awake	13/54 (24.1%)		
Asleep	23/36 (63.9%)		
*N* electrodes		0.3956*	0.4–31.9
1	1/6 (16.7%)		
2	35/84 (41.7%)		
Directional electrodes		< 0.0001*	2.1–637.3
yes	36/72 (50%)		
No	0/18 (0%)		
Lead Brand		0.0001^+^	
Medtronic	0/18 (0%)		0.0–0.6 (Medtronic vs. Boston Scientific)
Boston Scientific	25/56 (44.6%)		0.8–8.9 (Boston Scientific vs. Abbott/StJude)
Abbott/St Jude	11/16 (68.8%)		3.9–1534.1 (Medtronic vs. Abbott/StJude)
*N* MER tracks		0.0171*	1.3–8.9
1–2	13/50 (26%)		
> 2	17/31 (54.8%)		
Surgery Time (min)	276 (120–405) vs. 262 (150–488) in no edema	0.4695^#^	-50.0–28.0

CI, confidence interval; GPi, internal globus pallidus; MER, microelectrode recording; STN, subthalamic nucleus; VIM, ventro-intermedius nucleus of the thalamus

*, Fisher Exact test. ^+^, Chi-square test. ^#^, Mann-Whitney U test

On multivariable analysis (Table 5), awake surgery (*p* = 0.0451) was independently correlated to a reduced edema risk. The number of MER tracks showed a trend to an increased edema risk (*p* = 0.0504). The use of directional electrodes, instead, had no independent predictive value for post-operative edema.

**Table 5:** Multivariable analysis of predictive factors for edema

Parameter	Odds Ratio	95% CI	*p*
Awake DBS surgery	0.334	0.114–0.976	0.0451
Directional electrodes	6.8*10^− 7^	0-NA	0.9757
n MER tracks	1.932	0.999–3.736	0.0504

DBS, deep brain stimulation; MER, microelectrode recording

## Discussion

### Evidence from the literature

The occurrence of peri-electrode edema after DBS surgery was first recognized in a work published in 2004 on 38 patients undergoing staged DBS [1]. Edema was detected on MRI in 15 of 38 patients and, importantly, in all 7 cases in which MRI was performed by 1 month after implantation as compared to late scans. Since then, many studies investigated peri-electrode edema after DBS surgery (Table 6) [3–15]. In a large study on 133 DBS patients, Englot et al. found postoperative edema on MRI in 6.3% implanted electrodes [3]. They report that the only significant difference between edema and no-edema cases was the timing of scan, since cases showing perielectrode edema were imaged significantly later after surgery than those not showing edema (mean 5.1 vs. 2.0 days). Similarly, Borellini et al. showed a high edema incidence on MRI scan performed 7–10 days after surgery in 19 patients, while among 154 post-operative CT scans edema was never detected before 3 days post-surgery [10]. The higher incidence of edema at later post-operative scans was also pointed out in a recent review with meta-analysis of the literature [26]. According to this meta-analysis, the pooled incidence of radiological perilead edema in literature was 35.8%, and that of symptomatic edema was 3.1%. Importantly, a clear correlation between radiologic edema and clinical symptoms was not demonstrated [10]: papers focused on symptomatic post-operative perielectrode edema show its benign course with no long-term sequelae [4, 6, 8, 12, 27].

**Table 6:** Literature review of studies investigating peri-electrode edema after DBS surgery for PD

Author (year)	*N* cases	Type of study	Main finding
Ryu et al., 2004 [1]	38	Retrospective	Peri-lead edema reported only in MRI performed within 3 months since surgery. Tissue reaction to surgery or immune reaction responsible of edema.
Englot et al., 2011 [3]	133	Retrospective	Incidence of edema 6.3%, supposed to be of vasogenic origin. Mostly unilateral and asymptomatic. Images positive for edema performing later than negative ones.
Kim et al., 2013 [4]	173	Retrospective	Edema incidence 3.2%, median time to onset 4 days. Suggested to origin from mechanical breakdown of the blood-brain barrier. Self-limiting disorder, no additional treatments needed.
de Cuba et al., 2016 [5]	12	Retrospective	Delayed-onset edema (from a few days to months after surgery). Mild and aspecific symptoms, self-limiting. Pathophysiology still unexplained.
Fenoy et al., 2017 [6]	145	Retrospective	Symptomatic acute/subacute post-operative peri-lead edema in 3.4% cases. Corticosteroid treatment suggested in these cases.
Fernández-Pajarín et al., 2017 [7]	229	Retrospective	Paper dealing with DBS complications in general. Three edema cases (0.9% incidence) reported, two of whom symptomatic.
Nazzaro et al., 2017 [8]	189	Retrospective	Delayed, symptomatic edema in 3% of implanted leads, more common in re-implantations (9%) compared to new implantations (2%). Edema timing: onset 3–8 days after surgery, 4 to 6-week course.
Whiting et al., 2018 [9]	102	Retrospective	Peri-lead edema overall incidence 14.7%, symptomatic edema incidence 6.9%. Late MR scan (2 months after surgery). No risk factors for edema identified at statistical analysis.
Borellini et al., 2018 [10]	19 154	Prospective Retrospective	Peri-lead edema in all MRIs performed between 7 and 20 days after surgery in prospective cohort (19 pts) and in no CT scans performed within 3 days after surgery in retrospective cohort (154 pts). Most edema cases asymptomatic.
Saitoh et al., 2019 [11]	15	Retrospective	Edema incidence 40%. Two types of peri-electrode edema, *(i)* limited edema in deep white matter; *(ii)* extensive edema in surface white matter.
Sharma et al., 2019 [12]	260	Retrospective	Symptomatic peri-lead edema incidence 4.1%. Onset after 5.8 days. Benign course without long-term sequelae.
Nolt et al., 2020 [13]	13	Prospective	Serial post-operative MRI: the first incidence of edema most often within the first 2 weeks, but 1 case presented at 10 weeks. Benign course, often asymptomatic.
Erdem et al., 2022 [14]	30	Retrospective	Overview of adverse effects after DBS surgery. Low incidence of peri-lead edema (2.1%), with benign course.
Nishiguchi et al., 2022 [15]	13	Retrospective	Edema incidence 77% at MR performed 6 days after DBS. Frontal peri-lead edema caused transient cognitive decline, while STN edema associated with altered motor function.

DBS, deep brain stimulation; MER, microelectrode recording; PD, Parkinson Disease

### Findings of the study

In our work, the overall incidence of post-DBS edema and that of symptomatic edema was 40% and 1.1%, respectively; those figures closely recall the pooled incidence in current literature [26]. However, differently from previous studies, we did not find any difference in the timing of postoperative CT scan between edema and no edema cases (Table 3). Concerning risk factors for edema onset, only surgery under general anesthesia was independently associated with increased risk of perilead edema, with the number of MER tracks showing a mere trend (Table 5). Notably, Englot et al. [3] did not find such correlations. The finding on asleep surgery is puzzling. In our series of asleep patients, postoperative edema was more frequently registered in patients operated without MER recording than in those operated with MER (75% vs. 60.7%, respectively). In patients operated without MER, inhalation anesthetics may have been used, which reportedly cause vasodilation, thus fostering brain edema [28]. Moreover, the direct implantation of the macroelectrode without the prior insertion of the microelectrode might cause a more abrupt brain trauma, again increasing edema risk. All these considerations could explain this unexpected finding. Regarding the number of MER traces, our single-track multipass technique, in which one microelectrode per time is inserted [18], could have played a role in limiting their impact on the onset of postoperative edema.

A novel issue addressed in this paper is the possible association between the use of directional leads and post-operative edema. Fricke et al. [29] reported 4 cases of symptomatic delayed-onset edema in a cohort of 31 patients implanted with directional electrodes, while no similar cases were registered in a parallel cohort of 31 traditional ring electrodes, suggesting a causal association. The mechanisms underlying such possible behavior are quite obscure: subtle differences in materials or in production techniques, or post-operative electrode rotation, were advocated [29]. In our series, the implant of directional electrodes was not independently associated with a higher risk of post-operative edema at multivariable analysis (Table 5). Whether this latter evidence rules out a causal role of directional electrodes in postoperative edema onset or reflects the different numerosity of directional vs. ring electrodes in our series, remains to be ascertained.

Most importantly, aside from these speculations, our study confirms the literature data regarding the benign nature of postoperative edema and the absence of indications to medical therapy in most instances. Thus, an attitude at minimizing the number of MER tracks, or at reconsidering the use of directional electrodes, in order to reduce the risk of postoperative brain edema, is not justified based on our and literature data. In particular, the optimization of positive MER recordings and of clinically meaningful intraoperative stimulations, which are of invaluable help in obtaining the correct electrode location, are an important concern during DBS procedure. The single-track multipass technique, in which one microelectrode per time is inserted, is in our opinion a good compromise between the need of obtaining satisfactory MER recordings and the need of avoiding multiple useless traces which can increase the risk of edema.

### Limitations of the study

Among the limitations of the current study, we acknowledge its retrospective nature and the timespan of patients enrollment, during which the improvement in CT images acquisition and elaboration technologies could have somewhat impacted on our ability to promptly detect postoperative edema. The exclusion of a significant proportion of patients due to lacking or poor-quality post-operative CT images, by reducing the sample numerosity, is another limitation. On the other hands, patients have been homogeneously treated at a large-volume center, thus reassuring on the generalizability of gathered data.

## Conclusion

Our data confirm that the risk of symptomatic edema in DBS for PD is very low regardless of clinical and surgical parameters. Based on our results, to minimize the risk of postoperative edema, an ideal DBS procedure should be conducted using an awake technique and performing as many MER tracks as deemed necessary, avoiding unnecessary traces. The use of directional leads was not independently associated with postoperative edema.

## Electronic supplementary material

Below is the link to the electronic supplementary material.

Supplementary Material 1

Supplementary Material 2

## Data Availability

The data supporting the findings of this study are available on request from the corresponding author.

## Abbreviations

DBS: Deep brain stimulation
GPi: Globus pallidus interna
MER: Microelectrode recording
PD: Parkinson Disease
STN: Subthalamic nucleus
TCI: Target Controlled Infusion
TIVA: Totally intravenous anesthesia
