# Supplementary material for: Risk factors and significance of post-operative edema in Parkinson Disease patients submitted to deep brain stimulation. A ten-year case series
Source: Neurol Sci. 2024 Sep 19;46(2):761–8. doi: 10.1007/s10072-024-07774-4 (PMC11772388; doi:10.1007/s10072-024-07774-4)
Supplement: Supplementary file 1 — Supplementary Material 1 [file 10072_2024_7774_MOESM1_ESM.pdf]

**Supplementary Table S1. Baseline Characteristics of the study patients (*n*=90)**

| **Feature** | **n (%)** |
|---|---|
| M:F | 57:33 (63.3 : 36.7) |
| Age (mean ± SD) | 57.4 ± 10.0 |
| Disease duration (mean ± SD) (y) | 12.3 ± 7.2 |
| Autoimmune disease | 8 (8.9) |
| Diabetes | 2 (2.2) |
| Hypertension | 17 (18.9) |
| Vascular diseases | 3 (3.3) |
