# Supplementary material for: Risk factors and significance of post-operative edema in Parkinson Disease patients submitted to deep brain stimulation. A ten-year case series
Source: Neurol Sci. 2024 Sep 19;46(2):761–8. doi: 10.1007/s10072-024-07774-4 (PMC11772388; doi:10.1007/s10072-024-07774-4)
Supplement: Supplementary file 2 — Supplementary Material 2 [file 10072_2024_7774_MOESM2_ESM.pdf]

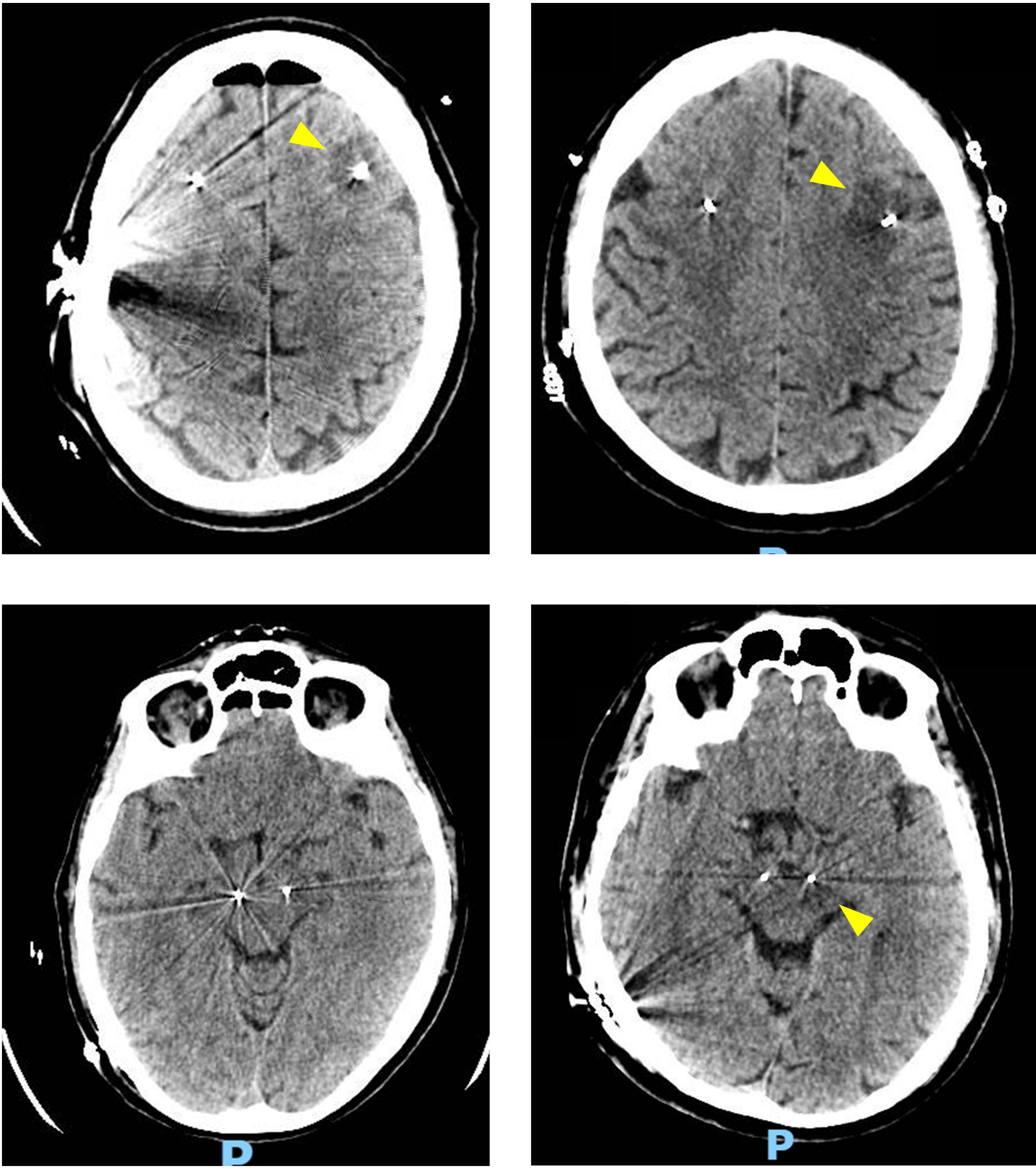

Supplementary Figure S1. Post-operative CT scan of patients operated on for bilateral STN DBS. *Left*, peri-lead edema limited to subcortical white matter. *Right*, whole electrode edema. Arrowheads point at edema.
